# Comparative transcriptome analysis revealed differential gene expression involved in wheat leaf senescence between stay-green and non-stay-green cultivars

**DOI:** 10.3389/fpls.2022.971927

**Published:** 2022-08-26

**Authors:** Qing Li, Huai Yang, Jingwei Guo, Qianglan Huang, Shengfu Zhong, Feiquan Tan, Tianheng Ren, Zhi Li, Chen Chen, Peigao Luo

**Affiliations:** ^1^Provincial Key Laboratory of Plant Breeding and Genetics, Sichuan Agricultural University, Chengdu, China; ^2^Department of Biology and Chemistry, Chongqing Industry and Trade Polytechnic, Chongqing, China; ^3^Insititue of Plant Protection, Sichuan Academy of Agricultural Sciences, Chengdu, China

**Keywords:** wheat (*Triticum aestivum* L.), leaf senescence, photosynthesis, chlorophyll, antioxidant enzyme, transcriptome

## Abstract

Breeders agree that leaf senescence is a favorable process for wheat seed yield improvement due to the remobilization of leaf nutrients. However, several studies have suggested that staying green may be an important strategy for further increasing wheat yields. In this study, we performed a comparative transcriptome analysis between wheat cultivars CN17 and CN19 after heading and also measured photosynthetic parameters, chlorophyll (Chl) contents, and antioxidant enzyme activities at various time points after heading. The physiological and biochemical indexes revealed that CN17 exhibited a functionally stay-green phenotype while CN19 did not. We identified a total of 24,585 and 34,410 differential expression genes between genotypes at two time-points and between time-points in two genotypes, respectively, and we also found that 3 (37.5%) genes for leaf senescence, 46 (100%) for photosynthesis – antenna protein, 33 (70.21%) for Chl metabolism and 34 (68%) for antioxidative enzyme activity were upregulated in CN17 compared with CN19 during leaf senescence, which could be regulated by the differential expression of *SAG39* (senescence-associated gene 39), while 22 (100%) genes for photosynthesis – antenna proteins, 6 (46.15%) for Chl metabolism and 12 (80%) for antioxidative enzyme activity were upregulated in CN17 compared with CN19 before the onset of leaf senescence. Here, we further clarified the expression profiles of genes associated with a functional stay-green phenotype. This information provides new insight into the mechanism underlying delayed leaf senescence and a new strategy for breeders to improve wheat yields.

## Introduction

From 1910 to 2005, the global net primary production (HANPP) doubled, but the global population increased fourfold (Krausmann et al., 2013). Thus, crop production must be further improved to meet the rising demand of the rapidly growing population (Fróna et al., 2019; Archana et al., 2021). As the most widely grown crop, wheat provides a great deal of calories and proteins for humans (Calderini et al., 2021). In the past, considerable improvements in wheat yield have been achieved through resistance improvement (Petersen et al., 2015; Ma et al., 2019), by the utilization of dwarfing genes (Thomas, 2017), and via morphological or type breeding (Pecetti and Annicchiarico, 1998). However, substantially increasing crop yields further via traditional breeding strategies has become increasingly difficult.

Leaf senescence, the final phase of leaf development, also has a large influence on crop yield (Guo et al., 2021). Hence, promoting the favorable onset and speed of leaf senescence could constitute a potential strategy to further improve crop yields. Some mutants referred to as “stay-green mutants,” which exhibit a normal green phenotype even after the onset of leaf senescence, have been identified in several species, such as rice (Fu et al., 2011; Archana et al., 2021), wheat (Christopher et al., 2008) and maize (Crafts-Brandner et al., 1984; Chibane et al., 2021). Stay-green genotypes have been classified into two categories, functional stay-green genotypes and cosmetic stay-green genotypes, the difference of which is based on whether they have effective photosynthesis during the process of leaf senescence (Hörtensteiner, 2009). Many breeders usually treat the stay-green phenotype as an obviously negative indictor of yield traits (Jiang et al., 2004; Antonietta et al., 2014), which could partly result from the stay-green phenotype of many mutants being only cosmetic rather than functional, and thus, stay-green mutants have been largely ignored.

However, functional stay-green mutants might be helpful to further increase crop yields because they can maintain photosynthesis during the later stages of growth, thus leading to the accumulation of increased amounts of photosynthetic products (Luo et al., 2006; Wang et al., 2021). Various studies have suggested that the cosmetic stay-green phenotype usually results from mutations in genes involved in leaf senescence, photosynthesis, chlorophyll (Chl) metabolism and antioxidative enzyme activity (Kura-Hotta et al., 1987; Zhang et al., 2016). Unlike for cosmetic stay-green genotypes, accurate identification of the phenotypes of functional stay-green genotypes is difficult mainly because there is continuous variation in the characteristics caused by the complex genetic makeup (Yoo et al., 2007) and in part because of the influences of other factors, especially those under field conditions, such as available water (Clifton-Brown et al., 2002), temperature (Kumari et al., 2007), drought (Borrell et al., 2000b) and diseases (Joshi et al., 2007; Li et al., 2015). Notably, because of these difficulties, developing functional stay-green commercial cultivars is the objective of only a few select breeders.

Fortunately, three functional commercial stay-green wheat cultivars, CN12, CN17 and CN18, with high yield and desirable agronomic traits were first developed via chromosome engineering, and they have been widely planted in southwestern China for approximately 20 years (Ren et al., 2003, 2004b). Previous studies have demonstrated that these cultivars are functional stay-green genotypes (Luo et al., 2006, 2009; Chen et al., 2010; Li et al., 2017), and their stay-green-related functions could be explained by the chloroplast ultrastructure regeneration hypothesis (Luo et al., 2013), of which the core idea was that the photosynthetic apparatus could be regenerated in the senescing leaf when the stacked grana thylakoid of PSII was retained in the early stage of leaf senescence. However, there is still no strong evidence at the gene expression level supporting this hypothesis, although some differentially expressed sequence tag (EST) sequences have been obtained by suppression subtractive hybridization (SSH; Luo et al., 2013). In addition, the non-stay-green control wheat cultivar MY11 employed in a previous study was also not a contemporaneous counterpart, and thus, the evidence from both gene expression and key pathways supporting the views of the chloroplast ultrastructure regeneration hypothesis are still unclear.

To identify the key genes and core metabolic pathways underlying delayed leaf senescence and provide more solid evidence for the stay-green function, the stay-green cultivar CN17 and its counterpart control, the non-stay-green cultivar CN19, were used for a comparative transcriptome analysis both before and after the onset of leaf senescence. In this study, we were interested in two classes of genes. The first genes (type I) were differentially expressed between CN19 and CN17 before leaf senescence (constitutive differential expression), with no difference in expression or opposite differential expression after leaf senescence; this differential expression accompanied a different tendency of gene expression changes from before leaf senescence to after leaf senescence in CN19 and CN17, respectively. The other class of genes (type II) were not differentially expressed between CN19 and CN17 before leaf senescence (non-constitutive differential expression), and the differential expression of these genes after leaf senescence occurred alongside a different tendency of expression changes from before leaf senescence to after leaf senescence in CN19 and CN17, respectively. In addition, photosynthetic parameters, Chl contents and antioxidant enzyme activities were also measured throughout the whole process.

## Materials and methods

### Plant materials and growing environment

The wheat cultivar CN17 (stay green) and the control cultivar CN19 (non-stay green), which were developed and released in 2003 (Ren et al., 2003, 2004a) and are high yielding, were employed in the research. CN17 was selected from the cross (91S-23/A302), while CN19 was selected from the cross (Q1104A/R935). CN17 and CN19 have similar leaf area, plant height, tiller number and developmental progress including both heading and flowering time because they are contemporaneous cultivars. These plant materials were sown by hand in clay soil on October 31st, 2016 at the agricultural research station of Sichuan Agricultural University (SAU) in the Wenjiang district (latitude 30°43′N, longitude 103°52′E), Chengdu city, Sichuan Province, China. That area has a temperate climate, and during the experimental period average air temperatures were approximately 12°C and the experiment received approximately 390 mm of rain. However, during the period from anthesis (March15th) to maturity, the average air temperatures were 20.5°C and 347 mm rainfall was received. During the growing season, 6 g and 14 g N ammonium nitrate were applied at the one node and heading stage, respectively. Additionally, diseases and pests were effectively controlled with fungicides and pesticides as required. The experiment was conducted in a randomized complete block design with three replications. Each block between two 0.5 m width alleys consisted of twelve 3 m-long rows and rows were spaced at 0.33 m apart, in which the interval between two adjacent plants was 0.15 m. Thirty-one plants of each cultivar with similar development progress were randomly selected from the inside and marked in field plots for subsequent experiments, of which 10 plants of each genotype were chosen for measuring the photosynthetic indexes at 0, 10, 20, 30, and 40 days after heading (DAH). Similarly, 15 plants of each genotype were used to determine the biochemical indexes at the same five time points, and six plants of each genotype (three biological replicates for each) at 20 DAH (before the onset of leaf senescence, the key time point for determining the stay-green phenotype or not) and 40 DAH (during leaf senescence, the key time point for determining the final difference) were selected for RNA sequencing (RNA-seq).

### Measurement of photosynthetic indexes

Photosynthetic indexes, including the net photosynthetic rate (*P*_*n*_), intercellular carbon dioxide concentration (*C*_*i*_), stomatal conductance (*G*_*s*_), the transpiration rate (*T*_*r*_), and the ratio of internal to atmospheric CO_2_ concentration (*C*_*i*_/*C*_*a*_), were measured with a Li-6400-02B photosynthetic apparatus (LI-COR, Lincoln, NE, United States) as previously described (Li et al., 2017). Measurements of the middle of flag leaves were made between 9.00 AM and 11.00 AM under the following conditions of 0.55–0.65 kPa vapor pressure deficit, 1000 μmol/m^2^/s actinic light intensity and 21–24°C air temperature. A measurement was logged 1 min after readings stabilized. The mean of three independent readings of each plant was taken to represent the value of the given plant, while the mean of ten plants was used as the parameter for the genotype.

### Determination of Chl contents

Flag leaves of each genotype (three replications) at each time point were used to determine the Chl contents according to the methods of Lichtenthaler (1987).

### Measurements of antioxidant enzyme activity

Flag leaves of CN17 and CN19 (three replications) collected every ten DAH were used for measuring the activity of antioxidant enzymes, including superoxide dismutase (SOD), catalase (CAT) and peroxidase (POD), according to the methods described by Beauchamp and Fridovich (1971); Pigeolet et al. (1990), and Bestwick et al. (1998), respectively.

### Data statistics and analysis

The data were analyzed via SPSS Statistics software version 23 (IBM, Armonk, NY, United States). The means were calculated, and the differences between two cultivars and at each of the five time points were compared by analysis of variance (ANOVA) and independent-sample *t*-tests.

### Ribonucleic acid extraction and RNA sequencing

In studied region, leaf senescence of wheat plants grown under natural field conditions usually occurs at 26–28 DAH, and therefore, three flag leaves of each genotype at 20 DAH were selected as representative samples before the onset of leaf senescence and at 40 DAH as representative samples after the onset of leaf senescence for RNA extraction. TRIzol reagent (Invitrogen, Carlsbad, CA, United States) was used to extract RNA in accordance with the manufacturer’s instructions. The RNA of the three flag leaves of each cultivar at each time point (constituting three biological replicates) was subjected to RNA-seq at Biomarker Technologies Corporation based on the Illumina HiSeq high-throughput sequencing platform. The raw data have been deposited in the BIG Data Center under accession number CRA007229.

### Alignment of RNA sequencing data, differential gene expression analysis and functional annotations

The raw sequence data were screened by removing adapter and primer sequences, reads containing more than 10% N bases and reads of low quality. Then, the cleaned were aligned to the wheat reference genome IWGSC RefSeq v1.0 by using TopHat2 software to obtain the location information on the reference genome, as well as information concerning the specific characteristics of the sample sequences (Kim et al., 2013). The expression level of all the genes was calculated using StringTie (Pertea et al., 2015) software. Then, DESeq was used to analyze the genes that were differentially expressed between samples (Wang et al., 2009). A fold change ≥ 2 and a false discovery rate (FDR) < 0.01 were used as screening criteria for the detection of differentially expressed genes (DEGs). The sequences of the obtained DEGs were aligned to the sequence information within the Kyoto Encyclopedia of Genes and Genomes (KEGG) database (Kanehisa and Goto, 2000), Pfam database (Finn et al., 2013), SwissProt database (Bairoch and Apweiler, 2000), and non-redundant (NR) database (Deng et al., 2006) to annotate gene functions via the BLAST method. KEGG pathway enrichment analysis of the DEGs was performed using the KEGG Orthology Based Annotation System (KOBAS; Mao et al., 2005). When we identified the constitutive DEGs truly responsible for leaf senescence prior to leaf senescence occurring, the genes with the same expression tendency in both CN19 and CN17 from before leaf senescence to after leaf senescence and the similar differential expression between these cultivars after leaf senescence were treated only as controls and were not included as true constitutive DEGs. Likewise, when we identified the induced DEGs truly responsible for leaf senescence, the genes with the same expression tendency in both CN19 and CN17 from before leaf senescence to after leaf senescence and with similar differential expression between them before leaf senescence were also treated only as controls and were not included in the genes with truly differential expression.

### Single-nucleotide polymorphisms of differentially expressed genes detected via comparison of expressed sequences

Genome Analysis Toolkit (GATK; McKenna et al., 2010) software was used to detect SNPs, and SnpEff (Cingolani et al., 2012) software was used to annotate the variants and predict the effects of mutations. Reliable SNPs were screened from among those with no more than 3 consecutive single-base mismatches in 35 bp and for whose the quality after sequential depth standardization was greater than 2.0. To identify the key genes responsible for the stay-green phenotype, the expressed sequences of the DEGs were compared between samples; the same SNPs in the reference genome and CN19 were used as controls. In addition, three biological replicates of each genotype at each time point for the same SNP were selected to avoid false-positive SNPs.

## Results

### Phenotypic observations

With respect to CN17, a stay-green wheat cultivar, a large portion of leaves remained green past 40 DAH, whereas the flag leaves of the control cultivar (CN19) began to turn yellow at 30–32 DAH, which indicated that the onset of leaf senescence in CN19 usually occurred before 30 DAH, which was a normal senescence timeframe. However, CN17 exhibited a delayed onset of senescence; the visible change from green to yellow occurred at 44–45 DAH, and all of its leaves were yellow at 52–54 DAH.

### Changes in photosynthetic parameters

**FIGURE 1:**
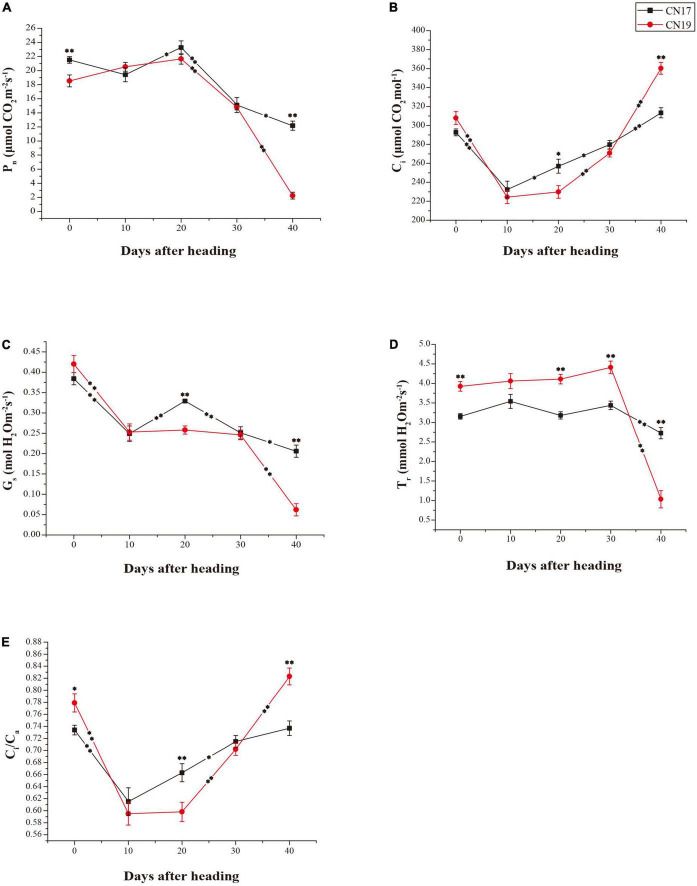
Differences in photosynthetic parameters between stay-green cultivar CN17 and non-stay-green cultivar CN19 after heading. **(A)** Net photosynthetic rate (*P*_*n*_); **(B)** Intercellular carbon dioxide concentration (*C*_*i*_); **(C)** Stomatal conductance (*G*_*s*_); **(D)** Transpiration rate (*T*_*r*_); **(E)** Ratio of internal to atmospheric CO_2_ concentration (*C*_*i*_/*C*_*a*_). The bars indicate the mean ± standard error (*n* = 10). * and ^**^ for significant differences at *P* ≤ 0.05 and *P* ≤ 0.01, respectively; the asterisks within the lines for significant differences between adjacent time points for the same wheat cultivar; the raised asterisks for significant differences between genotypes at each time point.

The *P*_*n*_ of CN17 was significantly higher than that of CN19 at both 0 and 40 DAH at the *P* = 0.01 level, and the change in the *P*_*n*_ exhibited the opposite tendency between CN17 and CN19 from 0 to 10 DAH, during which there was a decrease in the *P*_*n*_ in CN17 and an increase in CN19. In addition, the rate of decrease in the *P*_*n*_ was lower for CN17 than for CN19 from 30 to 40 DAH, and therefore, the *P*_*n*_ at 40 DAH was maintained at more than 60% and less than 10% of the *P*_*n*_ at 0 DAH in CN17 and CN19, respectively (Figure 1A).

The other photosynthetic parameters, including the *C*_*i*_, *G*_*s*_, *T*_*r*_ and *C*_*i*_/*C*_*a*_, of both CN17 and CN19 tended to change similarly during the whole process (Figures 1B–E), but there was usually a lower degree of change in these parameters (excluding *G*_*s*_) from 10 to 30 DAH in CN17 compared with CN19, especially from 30 to 40 DAH. Thus, compared with CN19, CN17 presented consequently higher *G*_*s*_ and *T*_*r*_ values as well as *P*_*n*_ values (Figures 1A,C,D) and lower *C*_*i*_ and *C*_*i*_/*C*_*a*_ values (Figures 1B,E) at 40 DAH.

### Chl components and contents

**FIGURE 2:**
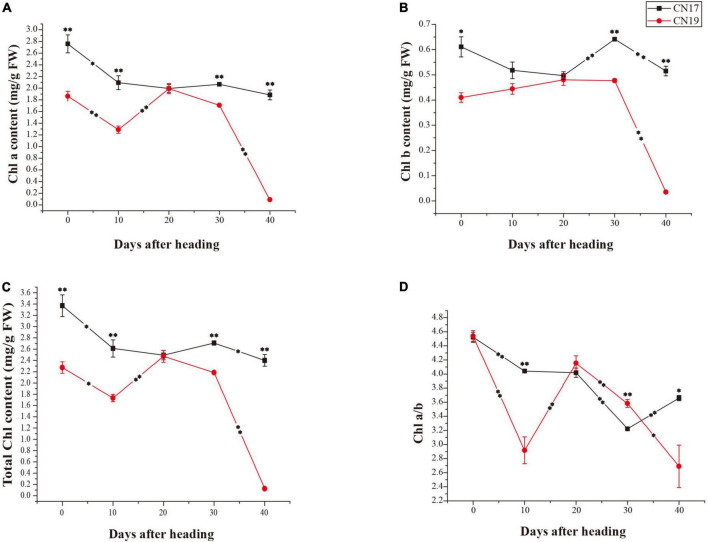
Differences in Chl contents in stay-green cultivar CN17 and non-stay-green cultivar CN19 after heading. **(A)** Chlorophyll a (Chl a) content; **(B)** Chlorophyll b (Chl b) content; **(C)** Total chlorophyll (Chl) content; **(D)** Ratio of Chl a to Chl b (Chl a/b). The bars indicate the mean ± standard error (*n* = 3). * and ^**^ for significant differences at *P* ≤ 0.05 and *P* ≤ 0.01, respectively; the asterisks within the lines for significant differences between adjacent time points for the same wheat cultivar; the raised asterisks for significant differences between genotypes at each time point; FW for fresh weight.

Compared with CN19, CN17 had significantly higher total Chl, chlorophyll a (Chl a) and chlorophyll b (Chl b) contents at all time points except for 20 DAH (Figures 2A–C). The contents of Chl a and Chl b in CN17 at 40 DAH were 68 and 84% of those at 0 DAH, respectively, while the contents of Chl a and Chl b in CN19 at 40 DAH were only 4.8 and 8.5% of those at 0 DAH (Figures 2A,B). Following a faster decrease, the Chl a/b ratio in CN19 significantly increased from 10 to 20 DAH, while a similar increase occurred during the later stage from 30 to 40 DAH in CN17 (Figure 2D).

### Antioxidant enzyme activity

**FIGURE 3:**
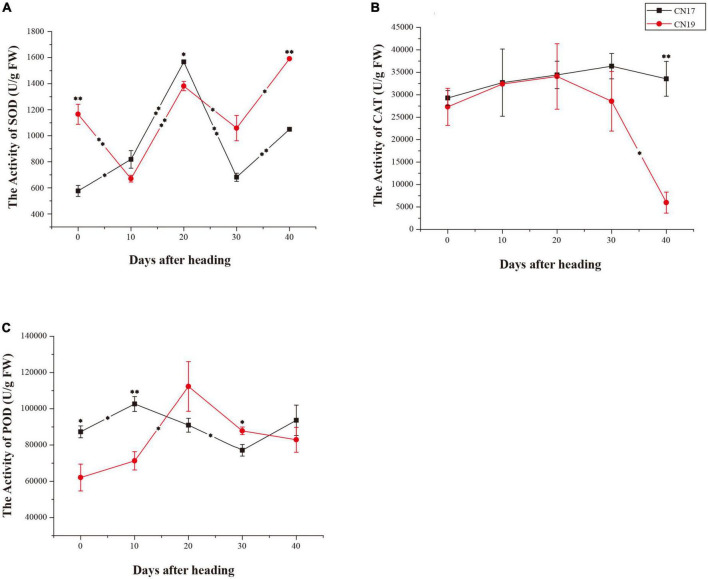
Differences in antioxidant enzyme activity in stay-green cultivar CN17 and non-stay-green cultivar CN19 after heading. **(A)** Superoxide dismutase (SOD); **(B)** Catalase (CAT); **(C)** Peroxidase (POD). The bars indicate the mean ± standard error (*n* = 3). * and ^**^ for significant differences at *P* ≤ 0.05 and *P* ≤ 0.01, respectively; the asterisks within the lines for significant differences between adjacent time points for the same wheat cultivar; the raised asterisks for significant differences between genotypes at each time point.

From 0 to 10 DAH, SOD activity significantly increased (*P* ≤ 0.05) in CN17 but significantly decreased (*P* ≤ 0.01) in CN19, and both CN19 and CN17 exhibited the same SOD activity change trend from 10 to 40 DAH, with a more rapid rate of change in CN17 than in CN19 from 20 to 40 DAH (Figure 3A). There was a similar change tendency of CAT activity in both CN19 and CN17 from 0 to 20 DAH (Figure 3B). After 20 DAH, the CAT activity increased from 20 to 30 DAH and slightly decreased from 30 to 40 DAH in CN17, while in CN19, it rapidly continuously decreased from 20 to 40 DAH (Figure 3B); thus, the CAT activity in CN17 was significantly higher than that in CN19 at 40 DAH (Figure 3B). The opposite change trend for POD activity occurred between CN17 and CN19 from 10 to 20 DAH and from 30 to 40 DAH, respectively (Figure 3C).

### Analysis of differentially expressed genes

On average, 9.58 Gb of data for each RNA sample were obtained from the leaves of both CN17 and CN19 at 20 and 40 DAH (Supplementary Table 1). Clean reads of each sample were mapped to the wheat reference genome, with an average alignment rate of approximately 80.44% (Supplementary Table 1). At 20 DAH, the number of genes differentially expressed between CN19 and CN17 was 8,160, with 4,057 upregulated and 4,103 downregulated in CN17 compared with CN19 (Table 1). At 40 DAH, the number of DEGs between them reached 16,425, of which 7,792 were upregulated and 8,633 were downregulated in CN17 (Table 1). Further analysis revealed that there were only 9,395 genes differentially expressed in CN17 between 20 and 40 DAH, while there were up to 25,015 in CN19 (Table 1).

**TABLE 1:** Comparison of DEGs in CN17 and CN19 at 20 DAH and 40 DAH.

DEG set	Number of DEGs	Upregulated	Downregulated
CN17–20 DAH vs. CN17–40 DAH	9,395	4,278	5,117
CN19–20 DAH vs. CN17–20 DAH	8,160	4,057	4,103
CN19–20 DAH vs. CN19–40 DAH	25,015	11,771	13,244
CN19–40 DAH vs. CN17–40 DAH	16,425	7,792	8,633

DEG, differentially expressed gene; DAH, Days after heading. N = 3 three biological replications.

### Classification of differentially expressed genes involved in the pathways possibly related to the stay-green phenotype

**FIGURE 4:**
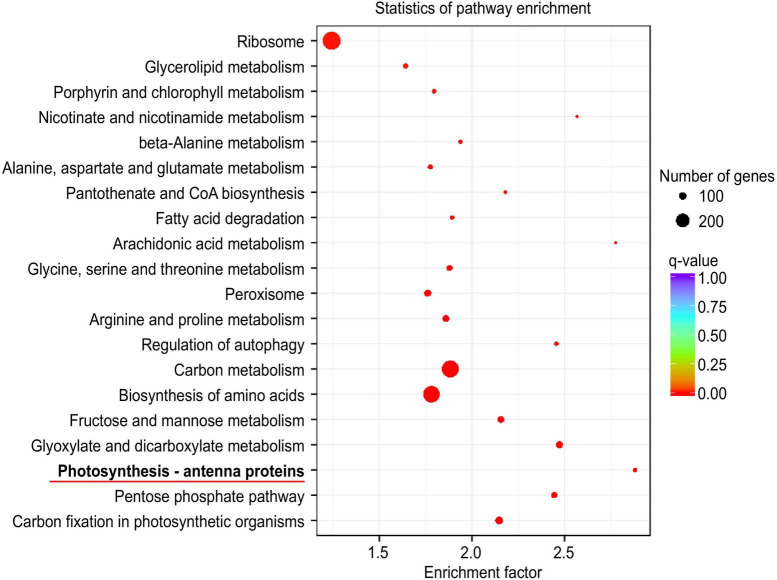
KEGG pathway enrichment analysis results between CN19 and CN17 at 40 DAH. Each circle in the figure represents a KEGG pathway. The vertical coordinate represents the name of the pathway, and the horizontal coordinate represents the enrichment factor. The larger the enrichment factor is, the more significant the enrichment level of DEGs in that pathway. The color of the circle represents the q value, which is the *P* value according to multiple hypothesis test corrections. The smaller the q value is, the more reliable the enrichment significance of the DEGs in that pathway. The size of the circle represents the number of genes enriched in the pathway. The larger the circle is, the more genes that are in that pathway.

**FIGURE 5:**
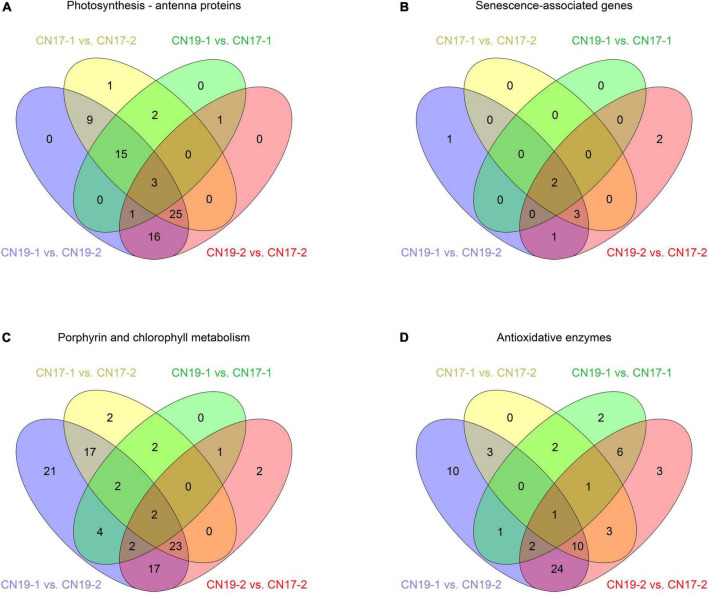
Number of DEGs between different comparison groups. **(A)** Photosynthesis – antenna protein; **(B)** Senescence-associated genes; **(C)** Porphyrin and Chl metabolism; **(D)** Antioxidative enzymes. The number 1 and 2 behind the cultivar stand for 20 DAH and 40 DAH, respectively.

A graph of the statistical analysis results of KEGG pathway enrichment between CN19 and CN17 at 40 DAH showed that photosynthesis – antenna proteins were ranked first (Figure 4), and further analysis revealed that there were 22 (green ellipse) and 46 (red ellipse) DEGs involved in this pathway between CN19 and CN17 at 20 and 40 DAH, respectively, of which only 5 DEGs were detected both before and after leaf senescence. Moreover, there were 55 (yellow ellipse) and 69 (blue ellipse) DEGs in CN17 and CN19, respectively, between 20 DAH and at 40 DAH, of which 52 were common to both cultivars (Figure 5A). These results suggested that the expression of genes involved in the photosynthesis pathway exhibited obvious differences between the stay-green and non-stay green plants, and therefore, the pathways closely related to photosynthesis, including leaf senescence, Chl metabolism and reactive oxygen species (ROS) metabolism, were also analyzed with similar methods.

A total of 9 DEGs were involved in leaf senescence, which is usually considered antagonistic to staying green, and there were only 2 (green ellipse) and 8 (red ellipse) genes differentially expressed between CN19 and CN17 at 20 and 40 DAH, respectively, of which the two genes differentially expressed between CN19 and CN17 at 20 DAH were also detected between them at 40 DAH (Figure 5B). In addition, there were 5 common DEGs following the leaf senescence process in CN17 and CN19 (Figure 5B).

Of the 95 DEGs involved in the Chl metabolism pathway, 13 (green ellipse) and 47 (red ellipse) DEGs were identified between CN19 and CN17 at 20 DAH and 40 DAH, respectively, and the number of DEGs between 20 and 40 DAH in CN17 (yellow ellipse) was lower than that in CN19 (blue ellipse; Figure 5C). Further analysis found that the genes differentially expressed between CN19 and CN17 were mainly involved in Chl biosynthesis and that there were more DEGs between these cultivars at 40 DAH than at 20 DAH (Supplementary Table 2). Similarly, among the 68 DEGs involved in antioxidative enzyme pathways, the number of DEGs between genotypes at 40 DAH (red ellipse) was greater than that at 20 DAH (green ellipse), whereas the number of genes that were involved in this pathway as well as Chl metabolism and that were differentially expressed before and after leaf senescence in CN17 (yellow ellipse) was also lower than that in CN19 (blue ellipse; Figure 5D).

### Identification of genes involved in the functional stay-green phenotype

Based on the foundational knowledge about wheat leaf senescence, two types of DEGs were interesting. Type I genes were differentially expressed between CN19 and CN17 before leaf senescence, and their differences in expression between genotypes after leaf senescence were not significant or opposite, which was possibly due to the different tendencies of expression changes during leaf development. Likewise, type II DEGs were identified between CN19 and CN17 after leaf senescence; these genes were not differentially expressed between genotypes before leaf senescence, which also resulted from the different responses to leaf senescence. In type I, 2, 6 and 3 genes were involved in photosynthesis – antenna proteins, Chl metabolism and antioxidative enzymes, respectively, and no DEGs involved in leaf senescence were classified as type I (Table 2). In contrast, 16, 17, 27 and 1 DEG involved in photosynthesis – antenna protein, Chl metabolism, antioxidative enzyme activity and leaf senescence, respectively, were classified as type II (Table 2). Interestingly, all 16 DEGs involved in photosynthesis – antenna proteins were found to encode Chl a-b binding protein.

**TABLE 2:** Numbers of DEGs of different classes in each pathway.

Expression pattern	Classes	Types of DEGs	CN19–20 DAH vs. CN17–20 DAH	CN19–40 DAH vs. CN17–40 DAH	CN17–20 DAH vs. CN17–40 DAH	CN19–20 DAH vs. CN19–40 DAH	Porphyrin and chlorophyll metabolism	Photosynthesis –antenna proteins	Antioxidative enzymes	Senescence-associated genes
							95	73	68	9
Constitutive expression	A1	DEGs with similar expression trends from before leaf senescence to after leaf senescence in both genotypes exhibited similar expression between genotypes both before and after leaf senescence	Down	Down	/	/	1		2	
			Up	Up	Down	Down	1	3	1	
			Up	Up	/	/		1	4	
			Down	Down	Up	Up				2
	A2	DEGs with similar expression trends from before leaf senescence to after leaf senescence in both genotypes with no differential expression between genotypes after leaf senescence	Down	/	Down	Down	1			
			Up	/	Down	Down	1	15		
			Up	/	/	/			2	
	A3	DEGs with different expression trends from before leaf senescence to after leaf senescence in both genotypes, with similar expression between genotypes both before and after leaf senescence	Down	Down	/	Down	1			
			Up	Up	/	Down	1	1	2	
			Up	Up	Up	Down	1			
			Up	Up	Up	/			1	
	**A4 (Type I)**	DEGs with different gene expression change trends from before leaf senescence to after leaf senescence in CN19 and CN17, with no differential expression or opposite expression between them after leaf senescence compared with before leaf senescence	Down	/	/	Down	4		1	
			Up	/	Down	/	2	2	2	
Non-constitutive expression	B1	DEGs with similar expression trends from before leaf senescence to after leaf senescence in both genotypes, with similar expression between genotypes both before and after leaf senescence	/	/	Down	Down	13	9	2	
			/	/	Up	Up	4		1	
	B2	DEGs with similar expression tendency from before leaf senescence to after leaf senescence in both genotypes exhibited differential expression between genotypes after leaf senescence, respectively	/	Up	Down	Down	14	25	6	
			/	Down	Up	Up	9		4	3
			/	Up	/	/	1		3	2
			/	Down	/	/	1			
	B3	DEGs with different expression trends from before leaf senescence to after leaf senescence in both genotypes, with no differential expression between genotypes after leaf senescence	/	/	/	Down	19		7	1
			/	/	/	Up	2		3	
			/	/	Down	/	2	1		
	**B4 (Type II)**	DEGs with different gene expression changetrends from before leaf senescence to after leaf senescence in CN19 and CN17, with differential expression between them after leaf senescence	/	Up	/	Down	15	16	15	1
			/	Down	/	Up	2		9	
			/	Down	Down	/			1	
			/	Up	Up	/			2	

Bold text indicates gene of interest. “Up” and “Down” represent upregulated and downregulated DEGs in the latter compared with the former, respectively.

Based on the sequences of the DEGs, we found 5 SNPs between CN19 and CN17. Only one synonymous SNP belonged to type I and was in *TraesCS1B01G059100* on chromosome 1B, encoding glutathione S-transferase. The other four non-synonymous SNPs (nsSNPs) were identified in DEGs of type II, and two nsSNPs were discovered in *TraesCS2B01G447300*, which encodes oxygen-dependent coproporphyrinogen-III oxidase, which participates in Chl biosynthesis, located at loci 640104224 and 640111777 of chromosome 2B. Similarly, a nsSNP was discovered in *TraesCS7B01G192500* on chromosome 7B, which is associated with photosynthesis – antenna proteins, and a SNP downstream from *TraesCS7A01G090400*, which encodes SOD, on chromosome 7A was also detected.

## Discussion

### CN17 exhibits a true functional stay-green phenotype and delayed leaf senescence

The advantages of delayed leaf senescence in terms of crop breeding include sustained photosynthesis during the later grain filling stage (Blandino et al., 2009). Previous studies have suggested that wheat leaf senescence usually occurs at approximately 21 days post-anthesis and at approximately 25 DAH; in CN17, a widely planted wheat cultivar with a 1BL/1RS translocated chromosome, leaf senescence is delayed by approximately 14 days and the senescence speed is slower compared with that needed for normal leaf senescence (Luo et al., 2006, 2009, 2013). In the present study, a decrease in *P*_*n*_ occurred in both CN17 and CN19 from 20 DAH, while the rate of decrease was lower for CN17 than for CN19, especially during the period from 30 to 40 DAH (Figure 1A). The parallel change between the *P*_*n*_ and *G*_*s*_ in CN17 from 10 to 40 DAH (Figures 1A,C) indicated that the photosynthetic ability of CN17 during senescence was mainly regulated by stomatal factors rather than by the activity of the photosynthetic apparatus. In addition, the lower *T*_*r*_ accompanying the equivalent or larger *G*_*s*_ of CN17 before 40 DAH (Figures 1C,D) suggested that, the water-use efficiency of CN17 is also high, the phenomenon of which is usually associated with drought resistance and the stay-green trait (Borrell et al., 2000a). Further comprehensive analysis of the changes in the *P*_*n*_, *G*_*s*_, and *C*_*i*_ suggested that the persistent increase in *C*_*i*_ in CN19 after 20 DAH possibly resulted mainly from the loss of photosynthetic ability rather than the decrease in *G*_*s*_ (Figures 1A–C). Consequently, the *P*_*n*_ of CN17 was significantly higher than that of CN19 at 40 DAH.

Previous studies have suggested that Chl b may be important for maintaining the stability of the light-harvesting Chl a/b-protein (LHCP) complex and the photosynthetic apparatus because it is primarily localized within the LHCP, while Chl a is a common component of all Chl-containing proteins (Bellemare et al., 1982; Luo et al., 2013). In this study, the total Chl, Chl a and Chl b contents were significantly higher in CN17 than in CN19 after leaf senescence (Figures 2A–C). This could provide some explanations in terms of apparatus for increased photosynthetic potential in CN17.

The hallmark of the functional stay-green phenotype is the prolonged high level of photosynthesis to provide increased amounts of carbohydrates, ultimately leading to the production of larger grains (Hörtensteiner, 2009). Previous studies have shown that CN17 produced larger grains than did the control MY11 (Luo et al., 2006), which was partially contributed to the smaller grains resulted from the variety degeneration of MY11, which was released in 1983. Some studies have shown that CN17 and CN19 have similar disease resistance and high yield (Luo et al., 2005, 2008, 2009). In this study, the rapid changes in the amounts of both Chl a and Chl b in CN17 (Figures 2A,B) ruled out the possibility that the stay-green phenotype was caused by the blocking of Chl degradation. Therefore, the delayed leaf senescence of CN17 was a true functional stay-green trait and would be helpful for further yield improvement; this phenotype is governed by a high photosynthetic ability that is possibly associated with both Chl content and antioxidant enzyme activity especially SOD and CAT after 20 DAH, which was also indirectly supported by the previous studies suggesting that both enhanced oxidative stress and reduced antioxidant activity may be a crucial factor to accelerate the process of leaf senescence (Prochazkova et al., 2001; Wang et al., 2016).

### The functional stay-green phenotype is associated with almost all pathways related to leaf senescence

The most noticeable difference between the functional stay-green phenotype and the cosmetic stay-green phenotype is whether these stay-green plants can maintain a high photosynthetic ability during leaf senescence (Hörtensteiner, 2009); the cosmetic stay-green phenotypes are related to only or mainly Chl degradation pathways (Pružinská et al., 2005; Barry, 2009).

In the present study, KEGG pathway enrichment analysis revealed that 46 genes differentially expressed between CN17 and CN19 after leaf senescence were mostly involved in photosynthesis – antenna proteins (Figures 4, 5A), and for 22 genes that were differentially expressed between the cultivars before leaf senescence, this pathway also ranked second (Figure 5A and Supplementary Figure 1). These findings indicated that the stay-green phenotype of CN17 could result from photosynthesis-related pathways such as leaf senescence, Chl biosynthesis and antioxidant enzyme activity because photosynthesis – antenna proteins play a key role in photosynthesis. We further identified 8, 47, and 50 genes that were differentially expressed between CN17 and CN19 at 40 DAH and involved in leaf senescence, porphyrin and Chl metabolism, and antioxidant enzymes, respectively (Figures 5B–D). In addition, the different change trends and values of the various photosynthetic parameters (Figure 1), Chl components and contents (Figure 2), and antioxidant enzyme activity (Figure 3) also indirectly suggested that these genes were differentially expressed.

There were also some genes that were involved in the abovementioned metabolic pathways and that were differentially expressed between the two genotypes at 20 DAH, but the number of DEGs and the change trend of some were obviously different. It is reasonable that the functional stay-green phenotype is associated with almost all the pathways related to leaf senescence, although we did not completely exclude the possibility of DEGs due to the differences between genotypes.

### Identification of key genes responsible for the functional stay-green phenotype

Previous studies have demonstrated that unlike the cosmetic stay-green phenotype, the functional stay-green phenotype usually involves multiple metabolic pathways and is controlled by many loci (Cook et al., 2021; Ren et al., 2022). The difference in leaf senescence between CN17 and CN19 is very conspicuous, especially in the later grain filling stage; therefore, despite their genetic background being different, as demonstrated by a pedigree analysis, these cultivars were used to detect DEGs (Li et al., 2017).

There are usually more DEGs detected when two genotypes have differences in their genetic background, and therefore, we need to pay more attention to screening and identifying the DEGs related to target traits. In this study, to identify the key genes responsible for the functional stay-green phenotype, we used the genes that were differentially expressed before leaf senescence as controls; these DEGs could have resulted from the differences in genetic background. At the same time, we further eliminated the common DEGs exhibiting similar expression change trends from 20 to 40 DAH between CN17 and CN19, which possibly resulted from the similar developmental processes of other traits, such as grain filling. Based on this idea, only type I and type II DEGs were interesting to us. There were 11 type I and 61 type II DEGs identified in the study (Table 2), which indicated that non-constitutive differential expression was the predominant mechanisms responsible for the stay-green phenotype, although we did not completely ignore the contribution of the constitutive DEGs involved in the stay-green phenotype.

Further analysis revealed that only one senescence-associated gene (*SAG39*) out of 72 interesting DEGs belonged to type II genes that we concerned (Table 2 and Supplementary Table 3); interestingly, a previous study confirmed that *SAG* could negatively regulate leaf senescence (Li et al., 2012). In this study, *SAG39* was upregulated in CN17 compared with CN19 at 40 DAH, and there was no difference in expression between cultivars at 20 DAH. In addition, *SAG39* was downregulated at 40 DAH compared with 20 DAH in CN19, while the expression change from before leaf senescence to after leaf senescence was not different in CN17 (Table 2 and Supplementary Table 3). These findings suggested that *SAG39* negatively regulates leaf senescence and delays leaf senescence mainly by regulating the stability of Chl-protein complexes and that the upregulated expression of *SAG39* in CN17 at 40 DAH possibly inhibited leaf senescence. Both 2 type I and all 16 type II DEGs involved in photosynthesis – antenna proteins encoded Chl a-b binding proteins (Table 2 and Supplementary Table 4); these proteins retained Chl in Chl-protein complexes and represent elegant control points of Chl degradation (Hörtensteiner, 2009) and regulation of ROS balance (Xu et al., 2012).

Interestingly, 17 type II DEGs were involved in Chl metabolism, of which 15 DEGs involved in the Chl biosynthesis were upregulated in CN17 compared with CN19 at 40 DAH; however, following the leaf senescence process, they were downregulated in CN19 (Table 2 and Supplementary Table 2). In contrast, the other two DEGs involved in Chl degradation were downregulated in CN17 compared with CN19 at 40 DAH, while following the leaf senescence process, they were upregulated in CN19 (Table 2 and Supplementary Table 2). One of these DEGs encodes the Chl b reductase NYC1, which directly regulates Chl degradation (Teng et al., 2021), and the other encodes heme oxygenase 1, which putatively and negatively regulates Chl biosynthesis by enhancing the other branch pathways of porphyrin and inhibiting the number of porphyrins available for Chl biosynthesis (Mahawar and Shekhawat, 2018). In addition, 6 type I DEGs involved in Chl metabolism were detected, of which two (Table 2 and Supplementary Table 2) DEGs encoding protochlorophyllide reductase A, were upregulated in CN17 compared with CN19 at 20 DAH, while following leaf senescence, they were downregulated in CN17, and the expression change showed no difference in CN19. The measured Chl component and content parameters were well agreed with the differential expression of the genes involved in Chl metabolism (Figure 2).

The changes in antioxidant enzyme activity after heading (Figure 3) indicated that the genes encoding these enzymes had very versatile role in leaf senescence (Table 2 and Supplementary Table 5), and the 3 type I and 27 type II DEGs exhibiting many patterns of expression changes between CN17 and CN19 from before leaf senescence to after leaf senescence (Table 2 and Supplementary Table 5) demonstrated the multiple roles of the DEGs in regulating leaf senescence. However, the four genes encoding CAT or CAT isozymes showed consistent expression changes; these genes were upregulated in CN17 compared with CN19 at 40 DAH, while following leaf senescence, CN19 was downregulated and CN17 did not differ in terms of expression (Table 2 and Supplementary Table 5). Therefore, the predominant role of genes encoding CAT or CAT isozymes positively regulated the stay-green phenotype, especially after leaf senescence, possibly by inhibiting the degradation of Chl-containing proteins (Zavaleta-Mancera et al., 2007) and by defending against cell oxidative damage from ROS (Lacan and Baccou, 1998).

### Single nucleotide polymorphisms in the key genes possibly enhance functionally stay-green phenotype

Usually, nsSNPs are important for producing new genes with functions that differ from those of old genes. In this study, we identified four nsSNPs from three genes. *TraesCS2B01G447300*, which encodes oxygen-dependent coproporphyrinogen-III oxidase, on 2B, *TraesCS7B01G192500*, which encodes Chl a-b binding protein 1B-21, on 7B, and a downstream SNP from *TraesCS7A01G090400*, which encodes SOD, on 7A were also detected.

*TraesCS2B01G447300* has 8 exons, within which two nsSNPs were located in exons 1 and 5, inducing a mutation from aspartic acid to asparagine and from alanine to aspartic acid, respectively, in the encoded protein. Coproporphyrinogen III oxidase (CPOX, EC 1.3.3.3) is a crucial enzyme in Chl biosynthesis, catalyzing coproporphyrinogen III to form protoporphyrinogen IX. Previous studies have indicated that mutations in the porphyrinogen III oxidase gene promoted early senescence in *Arabidopsis thaliana* (Guo et al., 2013).

There were 4 exons in *TraesCS7B01G192500*, in which a nsSNP was present in the first exon, causing a mutation from threonine to alanine in the encoded protein. This gene encodes a light-harvesting Chl protein complex (LHC) protein, commonly known as an antenna, which absorbs and transports light energy to the reaction center of the photosystem (Jansson, 1999). In addition, LHCs also play crucial roles in photoprotection, dissipating excess excitation energy as heat and reducing the formation of ROS, thus reducing light-induced oxidative damage (Crepin and Caffarri, 2015; Liguori et al., 2015). The remaining SNP was found in *TraesCS7A01G090400*, which encodes SOD and is involved in delaying senescence.

None of these three genes were differentially expressed before leaf senescence, but all three were substantially highly differentially expressed after leaf senescence between CN17 and CN19. In addition, different change tendencies during leaf senescence were detected between the genotypes. These findings indicated that these genes may indeed be differentially expressed as a result of the different responses to leaf senescence. These SNPs, especially the nsSNPs within the key genes, may contribute to the functional stay-green phenotype.

## Conclusion

**FIGURE 6:**
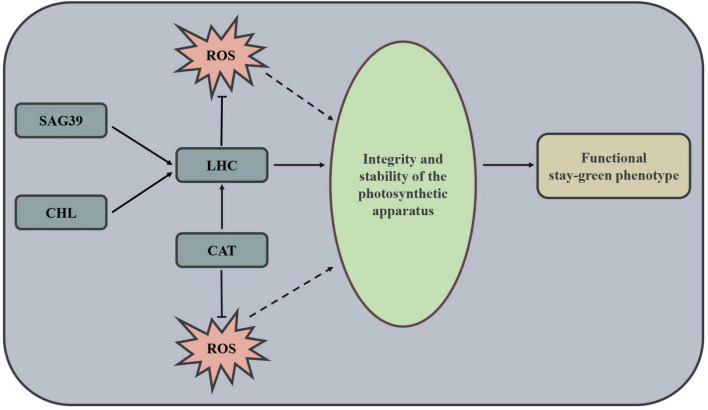
Mechanism governing the functional stay-green phenotype of wheat cultivar CN17. SAG, senescence-associated gene; CHL, chlorophyll; LHC, light-harvesting chlorophyll protein complex; CAT, Catalase; ROS, Reactive oxygen species.

Altogether, CN17 was confirmed to exhibit a true functional stay-green phenotype with high photosynthetic potential. This feature could mainly be attributed to the following processes or gene expression differences, as shown in Figure 6. Firstly, the upregulation of *SAG39* in CN17 could maintain the stability of chlorophyll-protein complexes by negatively regulating the leaf senescence process. Secondly, the upregulation of Chl synthesis-related genes and downregulation of Chl degradation-related genes may play a key role in maintaining high contents of chlorophyll and thus keeping the integrity of the chlorophyll-proteins. Thirdly, high expression of the genes encoding catalase or catalase isozyme could be helpful to maintain the perfect structure of chlorophyll-proteins.

## Data availability statement

The original contributions presented in this study are included in the article/Supplementary material. The sequencing data are publicly available at the BIG Data Center (https://ngdc.cncb.ac.cn) under accession number: CRA007229. Further inquiries can be directed to the corresponding author.

## Author contributions

PL, FT, and CC conceived the research. PL, TR, ZL, QL, and HY designed the experiments. QL, HY, JG, QH, and SZ conducted the experiments. QL, HY, JG, and PL analyzed the experimental data. QL, HY, and PL wrote the manuscript. All authors contributed to the article and approved the submitted version.
