# Supplementary material for: Comparative transcriptome analysis revealed differential gene expression involved in wheat leaf senescence between stay-green and non-stay-green cultivars
Source: Front Plant Sci. 2022 Aug 26;13:971927. doi: 10.3389/fpls.2022.971927 (PMC9459167; doi:10.3389/fpls.2022.971927)
Supplement: Supplementary file 1 [file Data_Sheet_1.ZIP › Caption for Supplementary Figure 1.DOCX]

Supplementary Material

# Supplementary Figures

**Supplementary Figure 1**. KEGG pathway enrichment analysis results between CN19 and CN17 at 20 DAH. Each circle in the figure represents a KEGG pathway. The vertical coordinate represents the name of the pathway, and the horizontal coordinate represents the enrichment factor. The larger the enrichment factor is, the more significant the enrichment level of DEGs in that pathway. The color of the circle represents the q value, which is the P value according to multiple hypothesis test corrections. The smaller the q value is, the more reliable the enrichment significance of the DEGs in that pathway. The size of the circle represents the number of genes enriched in the pathway. The larger the circle is, the more genes that are in that pathway.
