# Supplementary figures and images for: Comparative transcriptome analysis revealed differential gene expression involved in wheat leaf senescence between stay-green and non-stay-green cultivars
Source: Front Plant Sci. 2022 Aug 26;13:971927. doi: 10.3389/fpls.2022.971927 (PMC9459167; doi:10.3389/fpls.2022.971927)

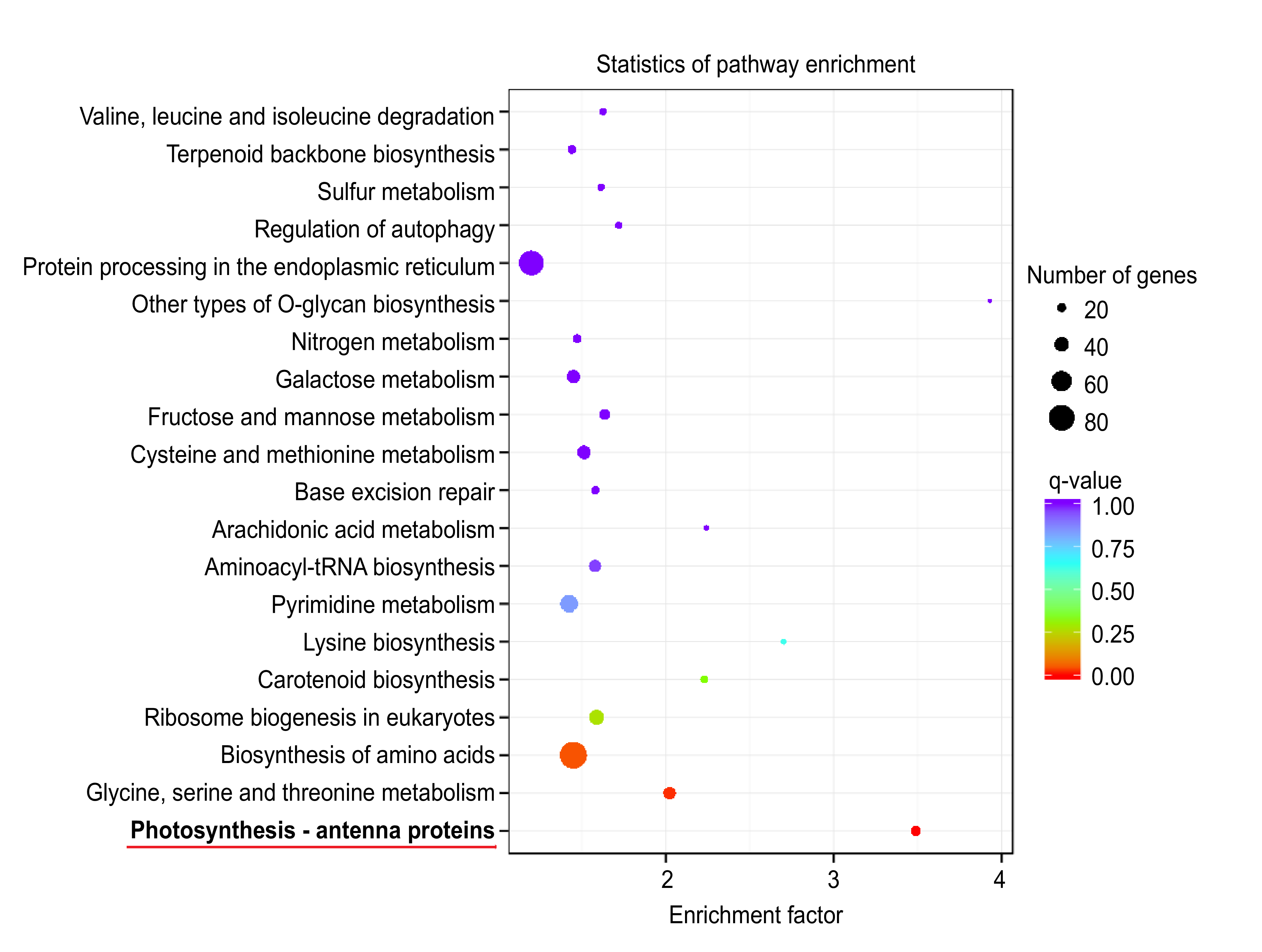

Supplement: Supplementary file 1 [file Data_Sheet_1.ZIP › Supplementary Figure 1.JPEG]
